# Carbohydrate Dependence During Prolonged, Intense Endurance Exercise

**DOI:** 10.1007/s40279-015-0400-1

**Published:** 2015-11-09

**Authors:** John A. Hawley, Jill J. Leckey

**Affiliations:** The Mary MacKillop Institute for Health Research, Centre for Exercise and Nutrition, Australian Catholic University, Locked Bag 4115, Fitzroy, VIC 3065 Australia; Research Institute for Sport and Exercise Sciences, Liverpool John Moores University, Liverpool, UK

## Abstract

A major goal of training to improve the performance of prolonged, continuous, endurance events lasting up to 3 h is to promote a range of physiological and metabolic adaptations that permit an athlete to work at both higher absolute and relative power outputs/speeds and delay the onset of fatigue (i.e., a decline in exercise intensity). To meet these goals, competitive endurance athletes undertake a prodigious volume of training, with a large proportion performed at intensities that are close to or faster than race pace and highly dependent on carbohydrate (CHO)-based fuels to sustain rates of muscle energy production [i.e., match rates of adenosine triphosphate (ATP) hydrolysis with rates of resynthesis]. Consequently, to sustain muscle energy reserves and meet the daily demands of training sessions, competitive athletes freely select CHO-rich diets. Despite renewed interest in high-fat, low-CHO diets for endurance sport, fat-rich diets do not improve training capacity or performance, but directly impair rates of muscle glycogenolysis and energy flux, limiting high-intensity ATP production. When highly trained athletes compete in endurance events lasting up to 3 h, CHO-, not fat-based fuels are the predominant fuel for the working muscles and CHO, not fat, availability becomes rate limiting for performance.

## Introduction and Background

The major metabolic consequences of the adaptations of skeletal muscle to endurance training are a slower utilization of carbohydrate (CHO)-based fuels (muscle and liver glycogen, blood glucose and muscle, blood and liver lactate), a greater reliance on fat-based fuels [adipose and intramuscular triglycerides (TGs), blood-borne free fatty acids (FFAs) and TGs] and less lactate production during low- to moderate-intensity exercise [i.e., 45–65 % of maximal oxygen uptake (*V*O_2max_)]. These adaptations, in part, underpin the substantial increases in submaximal exercise capacity observed following endurance training. Accordingly, many athletes and coaches steadfastly believe that fat plays an important role while training for and competing in endurance events lasting up to 3 h and that training and/or nutritional strategies that “spare” CHO-based fuels and enhance the oxidation of fat-based fuels will improve exercise capacity. In this review, we demonstrate that rates of fat oxidation over a wide range of speeds/power outputs are not substantially altered after endurance training when exercise is undertaken at the same relative intensity. This is because a major goal of training for performance enhancement is to promote skeletal muscle adaptations that allow an athlete to work at both higher absolute and relative power outputs/speeds, becoming more, rather than less reliant, on CHO-based fuels. In support of this contention, we show that competitive endurance athletes train and race at intensities that are highly dependent on CHO for muscle contraction. We also demonstrate that fat-rich diets do not “spare” CHO (i.e., muscle glycogen) or improve training capacity or performance, but rather directly impair the high rates of muscle glycogenolysis that are a necessary prerequisite for successful endurance performance. Finally, we propose that competitive athletes freely select CHO-rich diets because this strategy is essential to sustain muscle energy reserves and meet the daily demands of strenuous high-intensity endurance training. Using these independent but related lines of evidence, we demonstrate that athletes training for and competing in endurance events lasting up to 3 h are CHO dependent.

## Effects of Endurance Training on Patterns of Substrate Oxidation

It has become widely accepted that after a period of endurance exercise training, the non-protein respiratory exchange ratio [RER, volume of carbon dioxide production/volume of oxygen uptake (*V*CO_2_/*V*O_2_)] is lower, muscle glycogenolysis is reduced and fatty acid (FA) oxidation is greater compared with before training at a given exercise intensity. The phenomenon of increased fat utilization and CHO “sparing” in response to endurance training was first observed in early investigations using solely RER measures as an indirect estimate of whole-body substrate use [1]. It was not until the reintroduction of the percutaneous biopsy technique into exercise physiology that direct evidence of a training-induced muscle glycogen sparing effect was verified in humans [1, 2]. In recent years, indirect calorimetry in combination with isotopic tracer techniques and/or direct measures of substrate utilization from serial biopsies have been used to evaluate the regulation of endogenous fat and CHO metabolism in relation to exercise of varying intensities and a number of nutritional interventions [3–7]. Using a combination of these approaches, a large body of experimental evidence supports the overall interpretation that endurance training reduces the amount of CHO-based fuels oxidized during submaximal exercise, while the contribution from fat-based fuels to total energy expenditure increases [1, 8, 9].

While these general conclusions are valid, several caveats need to be considered within the framework of this paradigm. First, in the overwhelming majority of studies, subjects have only been tested at the same absolute (pre-training) work rate. Thus, even after short-term (i.e., 2- to 12-week) training interventions that typically improve *V*O_2max_ by 10–15 %, the relative intensity of exercise (as a proportion of the new, higher *V*O_2max_) is usually 10 % lower post-training. Not surprisingly, under these experimental conditions, rates of fat oxidation are always higher. Remarkably, few studies have tested subjects at both the same absolute and relative exercise intensities after a period of endurance training. Second, most studies employ previously untrained, predominantly male subjects to investigate the effects of short-term training interventions on patterns of substrate use. While there are major physiological and metabolic changes induced by the implementation of endurance training regimens in previously sedentary subjects, the results from these investigations bear little relevance to well-trained athletes with a history of many years of training. Third, subjects are frequently tested at a single submaximal work rate before and after a training intervention, mostly during exercise of low or moderate intensity (i.e., <65 % *V*O_2max_) and seldom at higher work rates at which fat oxidation would be minimal [10]. Finally, in the majority of studies, subjects are tested after a 10- to 12-h overnight fast. Notwithstanding the fact that competitive athletes are unlikely to commence the majority of training sessions and/or races with low CHO availability, such conditions would be expected to increase the contribution of fat-based fuels to total energy requirements, at least during exercise of low-to moderate-intensity.

Experimental evidence to support the contention that prior endurance training has little effect on patterns of substrate utilization and that both untrained and highly trained individuals are CHO dependent at high exercise intensities comes from the work of Bergman and colleagues [4, 11]. In one study, these workers trained nine male subjects for 9 weeks and measured whole-body RER, leg respiratory exchange quotient (RQ), tracer-derived measures of FFA fractional extraction and muscle TG utilization during 1 h of cycling at two exercise intensities before (45 and 65 % of *V*O_2max_) and after training (65 % of pre-training *V*O_2max_, the same absolute intensity, and 65 % of post-training *V*O_2max_, the same relative intensity). The training program was successful in promoting significant metabolic adaptations including a 15 % increase in *V*O_2max_. When subjects were tested at the same absolute (pre-training) intensity (i.e., 65 % of pre-training *V*O_2max_, representing 54 % of the post-training *V*O_2max_), there were increases in rates of whole-body fat oxidation (a decrease in RER from 0.96 to 0.93). However, when tested at the same relative intensity (65 % of pre- and post-training *V*O_2max_), RER values (0.95), leg RQ (0.98), net FFA uptake and muscle TG utilization were not different. While the absolute amount of CHO- and fat-based fuels increased, these data demonstrate that the balance of substrate utilization was unaffected by prior endurance training and that CHO-derived energy sources are the major fuel source for working muscle even during 1 h of moderate-intensity exercise [4].

In a second study, Bergman and Brooks [11] evaluated the interaction of training status and pre-exercise nutritional state on rates of substrate oxidation during graded cycling exercise. RER values were significantly lower in well-trained compared with untrained individuals during low- (22 % of *V*O_2max_) and moderate-intensity (40 % of *V*O_2max_) cycling when fasted and also during moderate-intensity exercise when fed or fasted. However, there was no training effect (i.e., lower RER values), nor any training-nutrient interaction at higher exercise intensities (60 and 75 % of *V*O_2max_). These data demonstrate that because athletes train and compete at exercise intensities >40 % of *V*O_2max_, they will not oxidize a greater proportion of fat substrates compared with untrained subjects, regardless of nutritional state. Taken collectively, these results [4, 11] demonstrate that the balance of substrate utilization is unaffected by prior endurance training and even during moderate-intensity exercise CHO-derived energy sources are the main fuel source for working muscle. Furthermore, at exercise intensities >60 % of *V*O_2max_, the relative power output is more important in determining the balance of substrate oxidation than either training or nutritional status. These observations clearly show that when evaluating the effect of prior exercise training on patterns of substrate utilization, it is critical that the data be placed within the context of the exercise testing paradigm [12].

## Endurance Athletes Train and Race at Intensities That Are Carbohydrate Dependent

A major goal of endurance training for the competitive athlete is to promote physiological and metabolic adaptations that increase the ability to sustain the highest average power output or speed of movement for a given distance [13–15]. In this regard, endurance training results in an increase in athletes’ *V*O_2max_ and also the fractional utilization of that (higher) aerobic capacity that can be sustained during training and competition [16]. While the absolute rates of oxidation of all classes of energy substrates increase after training, CHO-based fuels become the predominant energy source for trained muscle when exercise intensities are >60 % of peak oxygen uptake (*V*O_2peak_). This is because the balance of substrate oxidation at any time during exercise is a function of training-induced adaptations (which promote CHO oxidation) and endurance training-induced adaptations (which promote lipid oxidation) [12].

Direct measures of rates of fuel utilization during field-based training or competition are scarce particularly in highly trained athletes. Furthermore, there are only a limited number of laboratory-based investigations that have determined rates of substrate oxidation at the high absolute power outputs/speeds and relative exercise intensities (i.e., >80 % of *V*O_2peak_) that can be sustained by athletes during training and racing [6, 7, 17, 18]. With regard to the metabolic demands of endurance cycling training, Stepto et al. [18] reported that during an interval session (comprising 8 × 5-min work bouts at 85 % of *V*O_2peak_, ~325 W), rates of CHO oxidation were ~315 μmol/kg body mass (BM)/min while rates of fat oxidation were ~tenfold lower at ~30 μmol/kg/min. During steady-state cycling at power outputs of 310–320 W (80–85 % of *V*O_2peak_) maintained for ~30 min, rates of CHO oxidation typically range from 300 to 350 μmol/kg/min, corresponding to RER values of between 0.91 and 0.97 [6, 7, 17]. Recent data from Boorsma et al. [19] in elite runners (*V*O_2peak_ 80 ± 5 mL/kg/min, two subjects were Olympians) clearly show CHO dependence when running at speeds typically undertaken by these athletes in training. Boorsma et al. [19] determined rates of substrate oxidation from RER measures in eight male 1500-m runners during low- (50 % *V*O_2peak_), moderate- (65 % *V*O_2peak_) and high-intensity (80 % *V*O_2peak_) treadmill running. For the entire group, RER values were 0.85, 0.89 and 0.92 when running at 50, 65 and 80 % of *V*O_2peak_. However, for the top three runners with the highest *V*O_2peak_ values (83.4 mL/kg/min), RER was greater (0.94) when running at 80 % of *V*O_2peak_. At this intensity (corresponding to a speed of 19 km/h, 3:09 min/km), CHO-based fuels contributed 81 % to the total energy cost of running (Boorsma and Spriet, personal communication).

With regard to laboratory-based measures of substrate utilization during simulated competition, an early investigation reported CHO dependence during long-distance running. In that investigation O’Brien et al. [20] had a group of “fast” or “slow” runners complete a treadmill marathon under conditions that would be expected to favor fat oxidation (i.e., non-CHO loaded, overnight fast, no exogenous CHO provision during exercise). The “fast” runners completed the marathon in 2 h 43 min while the “slow” runners finished in 3 h 30 min. Runners in the “fast” group sustained a significantly greater fractional utilization of aerobic capacity compared with the slow runners (75 vs. 65 % of *V*O_2max_; *P* < 0.05), resulting in average RER values that were markedly higher (0.99 vs. 0.90; *P* < 0.05). However, there was no significant difference between “fast” and “slow” runners in the total amount of CHO-based fuels oxidized; the higher rate of CHO oxidation in the “fast” group was compensated by a longer running time in the “slow” group such that the total CHO combusted was similar (757 vs. 688 g for “fast” and “slow” runners, respectively). The results of O’Brien et al. [20] and others [21, 22] clearly show CHO dependence during endurance running lasting up to 200 min.

Given that the current world record for the men’s marathon is 2 h 2 min 57 s (an average speed of 20.59 km/h, 2:55 min/km), it has been proposed that CHO oxidation may be the exclusive source of energy for the working muscles when racing at such velocities [23]. This premise, along with the notion that competitive athletes train at intensities that are CHO dependent, is underpinned by limited laboratory-based measures of substrate utilization collected from elite runners. Coetzer et al. [24] compared a range of physiological and metabolic measurements in the fastest nine white and 11 black South African middle- and long-distance runners at the time of investigation. These workers reported that while both groups had similar training volumes, black athletes completed more running at intensities >80 % of *V*O_2max_ (36 vs. 14 %). At this intensity (equivalent to a running speed of 17 km/h), RER values measured during treadmill testing were 0.94, indicating 81 % of energy from CHO-based fuels. The fractional utilization of *V*O_2max_ that could be sustained by black athletes was greater than that of white athletes such that at half-marathon pace (21 km/h), black athletes could sustain 90 % compared with 82 % of *V*O_2max_ for the white runners. When running at 21 km/h (2:51 min/km), RER values approached 1.0 (total reliance on CHO-based fuels). When exercising at such high intensities, the energy yield per given volume of oxygen is 5.2 % higher from CHO- than fat-based fuels (5.058 vs. 4.795 kcal, respectively) [25]. Indeed, an increase in RER from 0.97 to 1.00 results in a 0.73 % increase in energy yield per liter of O_2_ consumed, and since the relationship between *V*O_2_ and speed is linear, this could potentially increase running speed by 0.15 km/h and improve the current world marathon record by approximately 50 s.

With regard to the substrate demands of intense endurance cycling, we have recently obtained data for eight competitive cyclists during a series of simulated time trials (TTs) lasting 60, 90 and 120 min and ridden at ≥80 % of *V*O_2max_ (Torrens and Areta, personal communication). These data show CHO dependence for all TTs independent of duration (mean RER values 0.97, 0.96 and 0.94, mean rates of CHO oxidation 360, 317 and 308 μmol/kg/min for 60-, 90- and 120-min TTs, respectively). Moreover, Cole et al. [26] have reported that gross mechanical efficiency during prolonged (2-h) cycling is improved and the decrease in efficiency over time attenuated, following 3 days of a high- (70 % of energy) compared with both a moderate- (45 % of energy) and low-CHO (20 % of energy) diet. While further work with elite athletes is needed to determine the metabolic demands of training and racing, it is clear that bioenergetics of sustained, high-intensity endurance exercise is CHO- rather than fat-dependent and that CHO is a more efficient fuel for muscular contraction during intense endurance exercise.

## Altering Substrate Availability Markedly Alters Patterns of Substrate Utilization but Does Not Enhance Exercise Capacity/Performance

The concept of altering substrate availability to modify the pattern of fuel utilization during exercise dates back almost a century when Krogh and Lindhard [25] first reported that subjects placed on a high-fat, low-CHO diet for several days had lower RER values during submaximal cycling than when they consumed a CHO-rich diet. Since that time, many studies have manipulated lipid availability before or during exercise and reported increased rates of fat oxidation and a “sparing” of endogenous CHO reserves, although these effects fail to translate into improved exercise performance. The topic of high-fat diets and athletic performance is summarized in a companion paper in this supplement [27] and has previously been reviewed elsewhere [28, 29]. However, given the renewed interest in promoting high-fat diets for endurance sport [30, 31], it is necessary to provide unequivocal evidence to demonstrate that such diets are detrimental for training and racing in endurance events lasting several hours.

The first modern-day investigation to revive interest in the concept of high-fat diets for athletic performance was that of Phinney et al. [32]. These workers studied five well-trained cyclists who first consumed a “balanced diet” for 1 week [BAL; 35–50 kcal/kg/day, 1.75 g protein/kg/day with the remainder of energy coming from CHO (66 %) and fat (33 %)] followed by 28 days of an isoenergetic, high-fat, low-CHO diet (KETO; <20  g/day). Although subjects were requested to continue with their normal training throughout the study, no objective measures of the volume, intensity or frequency of sessions, or the subjective ratings of perceived exertion (RPE) were reported. Furthermore, no metabolic parameters were collected during this period. At the end of the dietary intervention period, exercise capacity was assessed by the time to volitional fatigue while cycling at 63 % of *V*O_2max_ and was not different between BAL and KETO (147 ± 13 vs. 151 ± 25 min; *P* = 0.9). However, the average RER during the submaximal ride to exhaustion declined from 0.83 to 0.72 after the KETO diet, and this increase in fat oxidation coincided with a threefold drop in glucose oxidation and a fourfold reduction in muscle glycogen utilization [32]. The preservation of submaximal exercise capability appears impressive until one examines the individual responses to the dietary interventions: two subjects performed worse after the KETO diet, one performed the same, while of the two subjects who did improve, one rode substantially longer (148 vs. 232 min) so as to markedly skew the mean time. Of note was that four of the five subjects had a decline in *V*O_2max_ after the KETO diet while RER values at the end of the maximal test dropped from 1.0 to <0.9 in four subjects.

With regard to the effects of high-fat diets on training capacity, Stepto et al. [18] reported that subjective RPE was significantly greater after just 4 days of a fat-rich diet compared with an isoenergetic high-CHO diet when well-trained cyclists/triathletes undertook a standardized laboratory-based bout of intense interval training. In that study [18], RPE during non-laboratory training was also greater for cycling and all “other training,” during the high-fat compared with the high-CHO diet. In an effort to assess the impact of dietary changes on training and daily life, Stepto et al. [18] administered the Profile of Moods State (POMS) inventory to their subjects at the end of each day of the intervention. The global POMS score was significantly greater after 4 days of the high-fat compared with the high-CHO diet, while the individual POMS score for fatigue was also higher at this same time point [18]. To the best of our knowledge, only one other study has administered the POMS inventory to athletes while consuming a high-fat diet and attempting to maintain their normal training schedule. In that investigation, Keith et al. [33] reported an elevation in total mood score but no difference for the fatigue component of the inventory when moderately trained female subjects (*V*O_2peak_ 55 mL/kg/min) consumed a high-fat, low-CHO diet versus a high-CHO diet for 7 days.

As noted previously [29], high-fat “ketogenic” diet strategies represent as much a low-CHO challenge (i.e., training in the face of low muscle glycogen availability) as a high-fat challenge (i.e., training with high fat availability) and during such interventions muscle (and possibly liver) glycogen content is dramatically reduced. As such, recent interventions have focused on nutritional and training approaches that optimize endogenous CHO stores while concurrently maximizing the capacity for fat oxidation during continuous, moderate-intensity exercise. Such “nutritional periodization” typically encompasses a short-term (i.e., 5–6 days) high fat diet (60–70 % of total energy intake) followed by 24–36 h of high-CHO intake (70–80 % of energy, “CHO restoration”). Despite the brevity of the fat-adaptation period compared with some previous studies [32, 34], ingestion of a high-fat diet and the associated increased availability of FFA induces substantially higher rates of fat oxidation and concomitant muscle glycogen “sparing” during prolonged, submaximal exercise compared with an isoenergetic high-CHO diet (for reviews see [27–29]). Higher rates of fat oxidation during exercise persist even under conditions in which CHO availability is increased (i.e., a high-CHO meal before exercise and/or ingesting CHO-containing solutions during exercise) [35]. Yet, despite these robust changes in the patterns of fuel utilization that favor fat oxidation and “spare” endogenous glycogen utilization, high-fat, low-CHO diet strategies do not provide any benefit to the performance of prolonged endurance exercise, nor do they enhance training capacity. This is partly because the high rates of CHO oxidation obligatory to sustain the absolute and relative work rates typically attained by well-trained athletes during both training and competition when consuming a high-CHO diet are accompanied by increased glycolytic flux that directly inhibits lipolysis and the consequent uptake and oxidation of long-chain FAs [36–38]. While fat metabolism is down-regulated in the face of increasing CHO flux/availability and when moving from moderate to intense exercise, a reciprocal relationship exists to demonstrate that CHO metabolism is down-regulated in the face of increased fat availability. This glycogen “sparing” effect was originally seen as a positive outcome of a high-fat diet, but is now recognized as a direct impairment to CHO metabolism and likely underpins some of the reductions in exercise capacity observed after high-fat feeding.

High-fat diets rapidly down-regulate the amount of the pyruvate dehydrogenase (PDH) protein in the active form (PDHa) found at rest [39, 40]. This down-regulation is accomplished by rapid up-regulation of the enzyme PDH kinase (PDK), which moves PDH to the inactive form. Collectively, these mechanisms decrease CHO oxidation in the face of sub-optimal CHO availability, a response partly mediated by a reduction in circulating insulin concentration and the increased FFA levels after high-fat feeding [40]. During submaximal exercise following fat adaptation [41] and also fat adaptation and CHO restoration [42], PDH activation is reduced both at rest and over a range of exercise intensities. In this regard, the results of Stellingwerff et al. [42] are important. These workers measured muscle PDH activity before, during and after 20 min of cycling at 70 % *V*O_2peak_ and before and after 1 min of maximal sprinting at 150 % peak power output. Estimations of muscle glycogenolysis from serial biopsies were made during the initial minute of submaximal exercise at 70 % *V*O_2peak_ and immediately before and after the 1-min sprint. Despite 1 day of a high-CHO diet following 5 days of fat adaptation, resting PDH activity was 50 % lower than when subjects consumed a high-CHO diet for 6 days. During the first minute of submaximal exercise at 70 % of *V*O_2peak_ (211 W), rates of muscle glycogenolysis were reduced after fat adaptation/CHO restoration compared with the high-CHO condition due to substantially less pyruvate oxidation (via PDH flux). Even during the maximal 1-min sprint (~502 W), rates of glycogenolysis were reduced following the high-fat diet treatment. The suppression of PDH activity and rates of muscle glycogenolysis following high-fat diets impacts directly on high-intensity exercise capacity. Following moderate- and high-intensity aerobic training, maximal PDH activity increases [43, 44] to support the high rates of CHO oxidation that are essential for work rates requiring >80 % of *V*O_2max_. Therefore, the persistence of down-regulated PDH activity following fat-adaptation strategies, even in the face of CHO restoration, suggests that such dietary interventions are not advisable in situations in which sustained high-intensity efforts are required. Direct evidence of a negative effect of a high-fat diet on performance comes from Havemann et al. [45] and has been extensively discussed elsewhere [27, 46]. Consistent with the inhibition of resting and exercise-induced PDH activity, Raper et al. [47] have recently reported slower *V*O_2_ kinetics following 6 days of a high-fat diet compared with a high-CHO diet. Perhaps the final word on the topic of high-fat diets should be afforded to Phinney et al. [32], who stated unambiguously three decades ago that “there is potential benefit in a keto-adapted state for athletes participating in prolonged endurance exercise over two or more days.” Somewhere, this message seems to have been lost in translation in application to typical Olympic sports!

## Competitive Endurance Athletes Freely Choose to Consume High-Carbohydrate, Not High-Fat Diets

Twenty years ago it was claimed “despite the recent intrusion of sports nutritionists dedicated to the promotion of high CHO diets, athletes do not eat such CHO-rich diets in training and have not increased their CHO intake over the past 50 years” [48]. With recent calls to abandon high-CHO in favor of high-fat diets [30, 31], it is appropriate to briefly scrutinize the dietary habits of highly trained endurance athletes and assess whether these practices have changed in the last five decades. Although it would be naïve to attribute direct cause and effect between dietary practices and training/performance outcomes, one might assume that the majority of athletes through either a trial and error approach or after seeking professional nutritional advice, voluntarily consume a diet that meets the energy requirements of daily training, is palatable and appetizing, minimizes gastrointestinal discomfort during training/racing, optimizes physical and mental performance, and enhances recovery. The basic premise underlying our viewpoint is that if it were advantageous to consume high-fat diets, the best athletes would be following such practices. They are not!

A comprehensive review of the available literature on the dietary practices of athletes was conducted in 2000 to address some of the challenges made around the self-chosen CHO intakes of athletes [49]. Male endurance athletes typically consumed daily CHO intakes of 5–7 g/kg BM/day for general training needs, with some evidence that this was a higher intake than observed among athletes from earlier studies [49]. Some investigations involving competition nutrition, periods of increased training or elite athletes such as the Kenyan distance runners [50] or Tour de France cyclists [51] have reported greater intakes of 7–12 g of CHO/kg/day for periods. These CHO intakes, and the concomitantly moderate intakes of fat, are in line with sport nutrition guidelines of the corresponding era [52]. It should be noted that in contrast to male endurance athletes, some females are less likely to achieve recommended CHO intake guidelines, mostly due to their lower relative energy intake [49]. Notwithstanding possible limitations of dietary survey techniques when assessing the adequacy of the dietary practices of athletes (i.e., potential errors caused by under-reporting or under-eating during the period of the survey), the available data clearly demonstrate that endurance athletes from the 1990s up to 2005 consumed diets high in CHO and low in fat.

Official dietary guidelines for athletes have evolved over the last few decades to better define the goals and targets for optimal CHO intake in training and competition. Such guidelines now promote the goal of “high CHO availability” (intake of CHO targeted to meet the specific substrate needs of training/competition) rather than absolute CHO intakes per se. Furthermore, such a goal is aligned to training sessions or events when optimal performance is required, and there is tacit acknowledgement that higher CHO intakes or high CHO availability may not be needed around other sessions [27, 53]. Indeed, there are evolving practices of dietary periodization whereby some sessions are deliberately undertaken with low CHO availability to promote training adaptations [54, 55]. It should be emphasized, however, that these strategies are implemented acutely, are periodized so as to make up a small proportion of the training program, are avoided when high-quality/intensity training outputs are required, and are generally not achieved via the intake of a high-fat diet [56–58]. The overriding philosophy of undertaking quality training and competition with high CHO availability, to both promote training adaptations and the use of CHO as a substrate for the brain and central nervous system while performing optimally, is preserved [53]. While data regarding how highly trained competitive endurance athletes implement such practices is unavailable, evidence supports the notion that these athletes freely select CHO-rich or CHO-periodized diets rather than fat-rich diets. Such a strategy is essential to sustain muscle energy reserves and meet the daily demands of strenuous endurance training sessions. Whether this is a “neuro-biological” phenomenon (i.e., trial and error) or, indeed, due to the persuasive powers of sports nutritionists and the sports beverage industry has been the topic of previous discourse [59].

## Summary and Directions for Further Research

The main purpose of training for the enhancement of performance of prolonged (up to 3 h), continuous, high-intensity endurance sport is to promote a range of physiological and metabolic adaptations that permit an athlete to work at both higher absolute and relative power outputs/speeds and delay the onset of fatigue. To meet these goals, competitive endurance athletes perform a large proportion of their daily training at intensities that are close to race pace and highly dependent on CHO-based fuels for muscle metabolism. Consequently, to sustain muscle energy reserves and meet the daily demands of strenuous endurance training sessions, competitive athletes freely select CHO-rich diets.

Despite renewed popular interest in high-fat, low-CHO diets for endurance sports, fat-rich diets do not “spare” CHO (i.e., muscle glycogen) or improve training capacity/performance but, instead, directly impair rates of muscle glycogenolysis and energy flux. This down-regulation of CHO metabolism underpins the reductions in high-intensity exercise capacity observed after high-fat feeding. Indeed, when highly trained athletes compete in endurance events lasting up to 3 h, CHO-, not fat-based, fuels are the predominant fuel for the working muscles and CHO, not fat, availability becomes rate limiting for performance.

We presently lack detailed information on the metabolic demands of the training practices of competitive endurance athletes. This applies to the fuel requirements of individual training sessions as well as the impact of undertaking several workouts a day and/or multiple sessions in different disciplines (i.e., triathletes). Longitudinal data collected throughout an entire competitive season or during specific periodized training blocks are also needed to assess whether the energy (i.e., CHO) intakes of athletes fluctuate in accordance with alterations in training volume and load. Recent technological advances in gathering personalized diet information via mobile phone applications should aid in this quest. There is also an absence of data on the fuel requirements of elite athletes during actual competition. While the practical difficulties inherent in obtaining such information are acknowledged, laboratory-based measures of substrate oxidation in sub-elite athletes at the work rates/intensities sustained by competitive athletes during actual races would provide valuable insight into the extreme bioenergetics required for success in endurance events. In the meantime, it seems prudent to continue to recommend that well-trained athletes training for and competing in high-intensity endurance events lasting up to 3 h consume a diet commensurate with their periodized training requirements, and ensure high CHO availability before and during major competitions.
